# Caffeine May Abrogate LPS-Induced Oxidative Stress and Neuroinflammation by Regulating Nrf2/TLR4 in Adult Mouse Brains

**DOI:** 10.3390/biom9110719

**Published:** 2019-11-08

**Authors:** Haroon Badshah, Muhammad Ikram, Waqar Ali, Sareer Ahmad, Jong Ryeal Hahm, Myeong Ok Kim

**Affiliations:** 1 Division of Life Sciences and Applied Life Science (BK 21plus), College of Natural Science, Gyeongsang National University, Jinju 52828, Korea; hbadshah@awkum.edu.pk (H.B.); qazafi417@gnu.ac.kr (M.I.); waqarali_93@gnu.ac.kr (W.A.); sareer_50@gnu.ac.kr (S.A.); 2 Department of Internal Medicine, College of Medicine, and Division of Endocrinology, Gyeongsang National University Hospital and Institute of Health Sciences, Gyeongsang National University, Jinju 52828, Korea; jrhahm@gnu.ac.kr

**Keywords:** LPS, oxidative stress, neurodegeneration, caffeine, neuroprotection

## Abstract

Herein, we assayed the antioxidant and anti-inflammatory potential of caffeine in a lipopolysaccharide (LPS)-injected mouse model of neurodegeneration and synaptic impairment. For this purpose, LPS was injected for two weeks on an alternate-day basis (250 µg/kg/i.p. for a total of seven doses), while caffeine was injected daily for four weeks (30 mg/kg/i.p/four weeks). According to our findings, there was a significant increase in the level of reactive oxygen species (ROS), as evaluated from the levels of lipid peroxidation (LPO) and ROS assays. Also, we evaluated the expression of nuclear factor erythroid-2-related factor 2 (Nrf2) and the enzyme hemeoxygenase 1 (HO-1) in the mouse groups and found reduced expression of Nrf2 and HO-1 in the LPS-treated mice brains, but they were markedly upregulated in the LPS + caffeine co-treated group. We also noted enhanced expression of toll-Like Receptor 4 (TLR4), phospho-nuclear factor kappa B (p-NF-kB), and phospho-c-Jun n-terminal kinase (p-JNK) in the LPS-treated mice brains, which was significantly reduced in the LPS + caffeine co-treated group. Moreover, we found enhanced expression of Bcl2-associated X, apoptosis regulator (Bax), and cleaved caspase-3, and reduced expression of B-cell lymphoma 2 (Bcl-2) in the LPS-treated group, which were markedly reversed in the LPS + caffeine co-treated group. Furthermore, we analyzed the expression of synaptic proteins in the treated groups and found a marked reduction in the expression of synaptic markers in the LPS-treated group; these were significantly upregulated in the LPS + caffeine co-treated group. In summary, we conclude that caffeine may inhibit LPS-induced oxidative stress, neuroinflammation, and synaptic dysfunction.

## 1. Introduction

Elevated level of oxidative stress is the main contributor to the induction of neuronal cell loss and cognitive dysfunctions. Oxidative stress is induced by an imbalanced redox state, involving either excessive production of reactive oxygen species (ROS) or dysfunction of the antioxidant system defense mechanisms [1]. The brain is the most vulnerable organ to the effects of ROS because of its high oxygen demand and abundance of peroxidation-susceptible lipid cells [2].

Previous studies have shown that oxidative stress plays a pivotal role in the progression of neurodegenerative diseases such as Alzheimer’s disease [3] and Parkinson’s disease [1]. Many animal models have been developed based on elevated levels of oxidative stress, such as those induced by amyloid-beta (Aβ), lipopolysaccharide (LPS), and D-galactose [3]. Here, we used the LPS-injected mouse model. Lipopolysaccharides are characteristic components of the cell wall of Gram-negative bacteria; they are not found in gram-positive bacteria. They are localized in the outer layer of the membrane and are, in non-encapsulated strains, expressed on the cell surface. They contribute to the integrity of the outer membrane and protect the cell against the action of bile salts and lipophilic antibiotics [4].

LPS has been shown to be responsible for the induction of neuroinflammation in in vivo and in vitro models [3]. LPS has also been shown to elevate the level of ROS, which may subsequently induce the expression of inflammatory mediators and genes [5]. A controlled ROS level is maintained by the endogenous defense mechanism, mainly by nuclear factor erythroid-2-related factor 2 (Nrf2) and its downstream enzymes, such as hemeoxygenase 1 (HO-1) [6]. Elevated levels of ROS are responsible for the induction of neuroinflammation, mainly by inducing the activation of some inflammatory receptors (toll-like receptor 4 (TLR4) and other genes (nuclear factor kappa B (NF-kB), tissue necrosis factor alpha (TNF-α) [7]. Moreover, elevated ROS levels are also responsible for the induction of apoptotic cell death by activating Bax and cleaved Caspase-3 and by downregulating the expression of Bcl-2 [8].

Activation of the Nrf2 pathway could hinder the progression of neuroinflammation, mitochondrial apoptosis, and, ultimately, neurodegeneration [9]. Since the Nrf2 pathway plays a critical role in inflammation, Nrf2 activators have become a potential therapeutic strategy for numerous disorders, such as inflammatory disorders [10]. However, there are only very few Nrf2 activators in clinics. Dimethyl fumarate, a potent Nrf2 activator, has been approved for the treatment of multiple sclerosis [11]. Similarly, other antioxidants and Nrf2 activators have shown promising health benefits. Being the main driver of neurodegeneration, studies have always been focused on reducing the level of oxidative stress or on scavenging the ROS produced in the brain [12]. The best candidate drugs, which may reduce elevated ROS levels, are natural compounds [1].

Many natural compounds have shown superior efficacy as antioxidants against elevated levels of ROS. These antioxidants have also shown promising inhibitory effects against elevated levels of neuroinflammatory mediators [13]. The main contributors that aid in neuroinflammation in the brain are the innate immune response; activated astrocytes, as shown by the elevated expression of glial fibrillary acidic proteins (GFAP), and microglia, represented by enhanced expression of calcium-binding adapter molecule 1 (Iba-1); and other inflammatory markers and cytokines (NF-kB, TNF-α). These inflammatory mediators may induce apoptotic cell death, as determined by analysis of the relative expressions of Bax, cleaved caspase-3, and Bcl-2 [14]. The neuroinflammation may be coupled with synaptic loss and cognitive functions [3]. There are some well-known markers of synaptic dysfunction, postsynaptic density protein 95 (PSD-95) and synaptosomal-associated protein 23, which play crucial roles in the smooth processing of synaptic functioning [15].

Caffeine is the most widely used psychoactive compound in the world. As an integral component of tea, soft drinks, and coffee, caffeine is the most often consumed methylxanthine. The per capita consumption of caffeine from tea and coffee in the United Kingdom, Sweden, and Finland is estimated to be between 100 and 400 mg per person per day; these are the countries with the top consumption [16]. Extensive research studies have highlighted the protective potential of caffeine against different models of neurodegeneration [17].

Using Western blot, immunofluorescence studies, and some biochemical assays, we investigated whether caffeine can inhibit the LPS-induced neurodegeneration by regulating oxidative stress, neuroinflammation, apoptotic cell death, and synaptic deficits.

## 2. Material and Methods

### 2.1. Reagents and Antibodies

Lipopolysaccharide and caffeine were purchased from Sigma-Aldrich Chemicals Company (St. Louis, MO, USA). The antibodies used in the Western blot and immunofluorescence studies were anti-Nrf2 (sc-722), anti-HO1 (sc-136,961), anti-synaptosomal-associated protein 23 (SNAP-23) sc-374,215), anti-PSD-95(sc-71,933), anti-TNF-α (sc-52,746), TLR4 (sc-16240), anti-Bax(sc-7480), anti-Bcl2 (sc-7382), anti-p-NF-kB (sc-36,548), anti-Iba-1 (sc-32,725), anti-Glial fibrillary acidic protein (GFAP; sc-33,673), and anti-β-actin (sc-7,778) (Santa Cruz Biotechnology, Dallas, TX, USA). In addition, the anti-Cleaved Caspase-3 #9664) antibodies were obtained from Cell Signaling Technology (Danvers, MA, USA). Other primary antibodies were diluted in 1× TBST (1:1000), and secondary anti-mouse horseradish peroxidase (HRP)-conjugated (Promega Ref#W402) and anti-rabbit HRP-conjugated (Promega Ref# W401) antibodies were diluted 1:10,000 in 1× TBST(Sigma, Fitchburg, WI, USA). For the confocal microscopic studies, secondary fluorescent antibodies goat anti-mouse (Ref# A11029) and goat anti-rabbit (Ref# 32732) diluted in 1× PBS were used.

### 2.2. Animal Grouping and Drug Treatment

The mice (C57BL/6N male mice), with an average weight of 23–25 g, *n* = 60, were randomly divided into three groups: Control, LPS, and LPS + caffeine (20 mice per group, 10 mice for Western blot, and 10 for Immunofluorescence evaluations). The mice were obtained from Samtako BioLabs, Ulsan, South Korea and were acclimatized for one week under a 12 h light and dark cycle at room temperature, with free access to food and water. Caffeine and LPS were dissolved in normal saline (0.9%). The first group (control) was administered normal saline (1 mL/kg/day/i.p.) for six weeks. The second group was injected with LPS (250 μg/kg) for the last two weeks of the experiment—a total of seven doses. The third group was treated (i.p.) with caffeine (3 mg/kg/day/i.p.) for six weeks along with LPS. A caffeine-alone group was not included as no unwanted effects of caffeine have been reported previously [13]. All the experiments were carried out according to the procedures of the animal ethics committee (IACUC) of the Division of Applied Life Sciences, Gyeongsang National University, South Korea (Approval ID: 125).

### 2.3. Protein Extraction from Mouse Brains

After completion of the respective treatments, the animals were anesthetized and euthanized, and the brain tissues were removed. The brain tissue was dissected on dry ice, stored at −74 °C, homogenized in phosphate-buffered extraction solution (PRO-PREPTM^TM^, iNtRON Biotechnology, Inc., Sungnam, South Korea), and centrifuged at 13,000 rpm for 20 min. Proteins from the tissues were collected as the clear supernatant portion of the centrifuged tissue and stored at −74 °C.

### 2.4. Immunofluorescence Staining

The fluorescence was conducted as mentioned previously [18,19]. The dried slides were washed with PBS (0.01 mM) for 10–12 min (two times), incubated with proteinase-k for 6 min, and blocked with normal serum (2% goat/rabbit, as suitable) in PBS. The slides were incubated with primary antibodies overnight, washed with PBS, and treated with tetramethylerhodamine isothiocyanate–fluorescein isothiocyanate (FITC)-labeled secondary antibodies (antirabbit and antimouse, as suitable) at room temperature for 2 h. Images were taken using a confocal laser microscope (FluoView FV 1000 MPE, Olympus, Tokyo, Japan). The integrated density, which represents the sum of the pixel values in an image, was used for the quantification of the staining intensity, and the fluorescence was evaluated using ImageJ software (wsr@nih.gov, https://imagej.nih.gov/ij).

### 2.5. Determination of Reactive Oxygen Species

The ROS assay is based on the oxidation of 2, 7-dichlorodihydrofluorescein diacetate (CAS 4091-99-0, Santa Cruz Biotechnology, Dallas, TX, USA) to 2, 7-dichlorofluorescein (DCF). The brain homogenates were diluted in ice-cold Lock’s buffer at 1:20 to yield a final concentration of 2.5 mg tissue/500 µL. The reaction mixture of Lock’s buffer (1 mL; pH 7.4), 0.2 mL of homogenate, and 10 mL of DCFH-DA (dichlorodihydrofluorescein diacetate) (5 mM) was nurtured at room temperature for 15 min to convert the DCFH-DA to the fluorescent DCF. The transformation of DCFH-DA to DCF was evaluated by using a spectrofluorimeter (Promega, Fitchburg, WI, USA) with emission at 530 nm and excitation at 484 nm. For the background fluorescence, matching blanks were used, as performed previously [3].

### 2.6. Determination of Lipid Peroxidation

Free malondialdehyde (MDA), which is an indicator of LPO, was evaluated in the tissue homogenates by using a lipid peroxidation (MDA) colorimetric/fluorometric assay kit (BioVision, San Francesco, CA, USA, Cat#739-100), according to instructions used previously [10].

### 2.7. Western Blot Analysis

The protein concentration was determined using a BioRad protein assay kit, CA, USA. An equal amount of protein was loaded in SDS-PAGE, as performed previously [18]. For the size determination, a pre-stained protein marker was loaded (7–200 kDa, GangNam stain, iNtRON Biotechnology) and was resolved electrophoretically. The proteins were transferred to a polyvinylidene difluoride (PVDF) membrane (Immobilon-PSQ, Transfer membrane, Merck Millipore, Burlington, MA, USA). After the successful transfer, the membranes were blocked in 5% Skim Milk. After that, the membranes were blocked with the respective primary antibodies. After washing, the membranes were treated with the respective secondary antibodies for 2 h. The immunoblots were visualized on X-ray films using a fluorescent detection reagent (EzWestLumiOne, ATTO, Tokyo, Japan), and the respective densities of the bands were evaluated using Image J software (version 1.50, NIH, https://imagej.nih.gov/ij/, Bethesda, MD, USA). Graphs were generated using GraphPad Prism 6 software.

### 2.8. Data Analysis

We performed one-way ANOVAs with Tukey’s posthoc testing to draw comparisons among the different groups. The data are presented as the “mean (SD)” of 10 mice per group and are representative of three independent experiments. The calculations and graphs were generated using Prism 6 software (GraphPad Software, San Diego, CA, USA). In the results presented, symbol “*” indicates significant difference from the vehicle treatment, while “#” indicates significant difference from the LPS-treated group. Adjusted values are marked for significance as follows: *, *p* < 0.05; #, *p* < 0.05.

## 3. Results

### 3.1. Caffeine Prevents LPS-Induced Oxidative Stress in Mouse Brain

**Figure 1:**
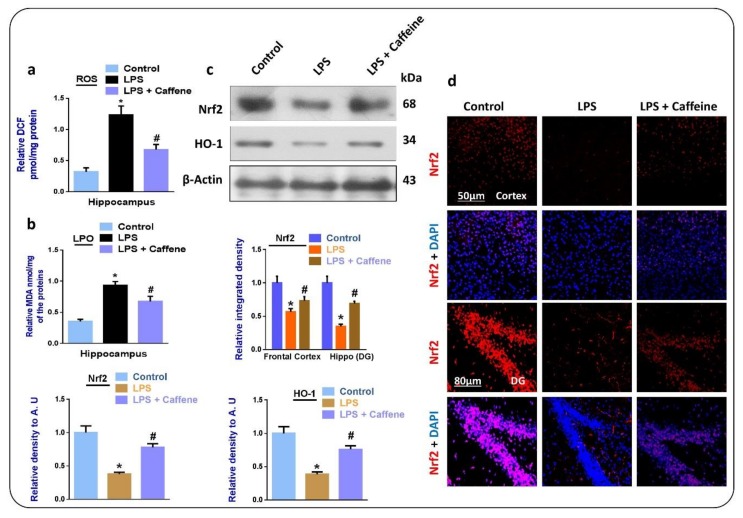
Caffeine inhibits lipopolysaccharide (LPS)-induced oxidative stress in mouse brain: (**a**,**b**) Results of reactive oxygen species (ROS) and lipid peroxidation (LPO) assays, respectively; (**c**) Western blot results of nuclear factor erythroid 2-related factor 2 (Nrf2) and hemeoxygenase 1 (HO-1), in the mouse brain; (**d**) confocal microscopic images of Nrf2 in the experimental groups, magnification 30×, scale bar 50 µm; * significantly different from the vehicle-treated control group, #, significantly different from the LPS-treated mouse group (*n* = 10 mice per group; number of experiments = 3; statistical analysis used was one-way ANOVA with Tukey’s posthoc test for comparisons among the different groups). Significance: * *p* < 0.05; # *p* < 0.05. DAPI: 4′, 6-diamidino-2-phenylindole. DG: Dentate gyrus.

To analyze the possible antioxidant effects of caffeine against LPS-induced oxidative stress, we evaluated the expressions of LPO and ROS in the brain homogenates of the experimental groups by way of the respective biochemical assays. According to our findings, there was enhanced expression of LPO and ROS in the LPS-injected mouse group (*n* = 5) compared to in the saline-injected control group. Interestingly, these effects were markedly reduced in the LPS + caffeine co-treated group (Figure 1a,b, respectively). We also evaluated the effects of caffeine against the specific endogenous ROS regulators and their downstream enzymes, such as Nrf2 and HO-1. According to our findings, there was a significant reduction in the expression of Nrf2 and HO-1, which were markedly upregulated in the LPS + caffeine co-treated group (Figure 1c). Moreover, according to the immunofluorescence analysis, there was a significant reduction in the expression of Nrf2 in the LPS-treated group, but it was markedly significantly upregulated in the LPS + caffeine co-treated group (Figure 1d).

### 3.2. Caffeine Suppresses LPS-Induced Activated Glial Cell Markers and Inflammatory Mediators in Mouse Brain

**Figure 2:**
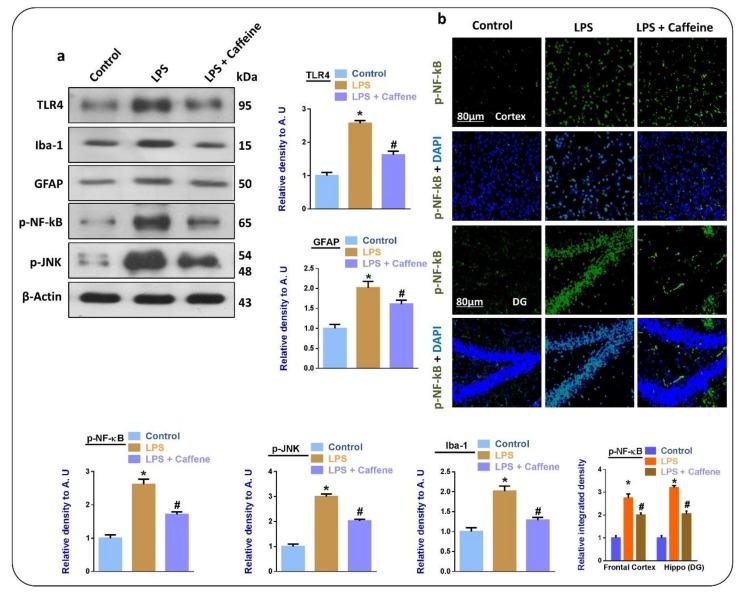
Caffeine inhibits LPS-induced glial cell neuroinflammatory mediators in mouse brain: (**a**) Western blot results of TLR4, GFAP, Iba-1, p-NF-kB, and p-JNK in the brains of the experimental mice (*n* = 10 mice per group; number of experiments = 3; statistical analysis used was one-way ANOVA with Tukey’s posthoc test for comparisons among the different groups); (**b**) confocal microscopic images of p-NF-kB in the experimental groups, magnification 30×, scale bar 80 µm; * significantly different from the vehicle-treated control group, #, significantly different from the LPS-treated mouse group. Significance: * *p* < 0.05; # *p* < 0.05. DAPI: 4′, 6-diamidino-2-phenylindole. DG: Dentate gyrus.

The innate immune receptor toll-like-receptor 4 (TLR4), localized on the surface of microglia [20], and activated glial cells are significant contributors to the release of inflammatory cytokines and neuroinflammation. Therefore, here we evaluated the expression of GFAP (a marker of activated astrocytes) and Iba-1 (a marker of activated microglia) in the experimental groups. According to our findings, there was a significant increase in the expression of TLR4, GFAP, and Iba-1 in the LPS-treated group compared to in the control group. Interestingly, these markers were markedly reduced in the LPS + caffeine co-treated group, as analyzed by Western blot analysis. Moreover, the expression levels of p-NF-kB and p-JNK were significantly higher in the LPS-treated mice but were reduced in the LPS + caffeine co-treated group (Figure 2a). Similarly, the immunofluorescence results also showed elevated expression of p-NF-kB in the LPS-treated mice brains (cortex and Hippocampus) but significantly reduced expression in the LPS + caffeine co-treated group (Figure 2b).

### 3.3. Caffeine Attenuates the LPS-Induced Release of Inflammatory Cytokines in Mouse Brain

**Figure 3:**
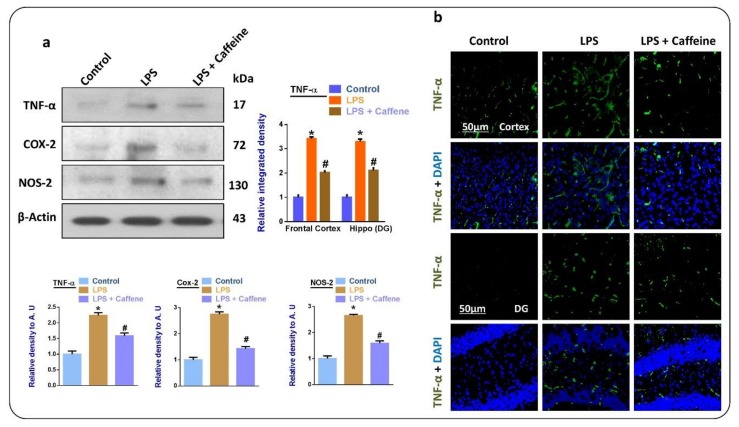
Caffeine inhibits the release of inflammatory cytokines in LPS-injected mice: (**a**) Western blot results of tissue necrosis factor α (TNF-α), nitric oxide Synthase 2 (NOS-2), and cyclooxygenase 2 (Cox-2) in the brains of the experimental mice (*n* = 10 mice per group; number of experiments = 3; statistical analysis used was one-way ANOVA with Tukey’s posthoc test for comparisons among the different groups); (**b**) immunofluorescence images of TNF-α in the experimental groups, magnification 30×, scale bar 50 µm; * significantly different from the vehicle-treated control group, # significantly different from the LPS-treated mice group. Significance: * *p* < 0.05; # *p* < 0.05. DAPI: 4′, 6-diamidino-2-phenylindole. DG: Dentate gyrus.

Inflammatory cytokines are prominent mediators in the further release of inflammatory cytokines such as tissue necrosis factor α (TNF-α), nitric oxide synthase 2 (NOS-2), and cyclooxygenase 2 (Cox-2). Here, according to our findings, there was enhanced expression of TNF-α, NOS-2, and Cox-2 in the LPS-injected mice group, while there was a significant reduction of these in the LPS + caffeine co-treated group (Figure 3a). The immunofluorescence results confirmed that there was enhanced expression of TNF-α in the frontal cortical and hippocampal (DG) sections of the LPS-injected mouse brains. Here, the TNF-α expression was significantly reduced with the administration of caffeine, compared to that in the LPS-injected mice (Figure 3b).

### 3.4. Caffeine Attenuates LPS-Induced Mitochondrial Apoptosis in Mouse Brains

**Figure 4:**
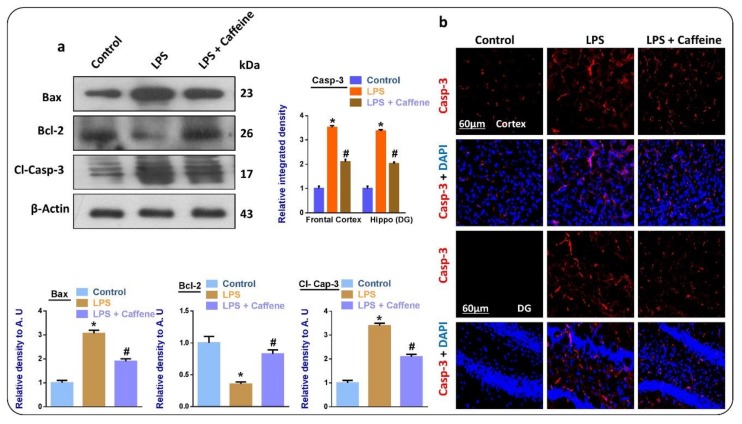
Caffeine inhibits mitochondrial apoptosis in the LPS-injected mice: (**a**) Western blot results of BCL2-associated X, apoptosis regulator (Bax), B-cell lymphoma 2 (Bcl-2), and cleaved Caspase-3 in the brains of the experimental mice (*n* = 10 mice per group; number of experiments = 3; statistical analysis used was one-way ANOVA with Tukey’s posthoc test for comparisons among the different groups); (**b**) immunofluorescence images of cleaved Caspase-3 in the experimental groups, magnification 30×, scale bar 60 µm; * significantly different from the vehicle-treated control group, # significantly different from the LPS-treated mice group. Significance: * *p* < 0.05; # *p* < 0.05. DAPI: 4′, 6-diamidino-2-phenylindole. DG: Dentate gyrus.

Next, we have evaluated another hallmark of neuronal cell loss—mitochondrial apoptosis. According to our findings, there was enhanced expression of BCL2-associated X, apoptosis regulator (BAX), and cleaved caspase-3 and reduced expression of B-cell lymphoma 2 (Bcl-2) in the LPS- injected mice. Interestingly these markers were markedly upregulated in the LPS + caffeine co-treated group (Figure 4a). The immunofluorescence results also supported the anti-apoptotic effects of caffeine against LPS as we analyzed the expression of cleaved caspase-3 in the experimental groups (Figure 4b).

### 3.5. Caffeine May Attenuate LPS-Induced Synaptic Dysfunction

**Figure 5:**
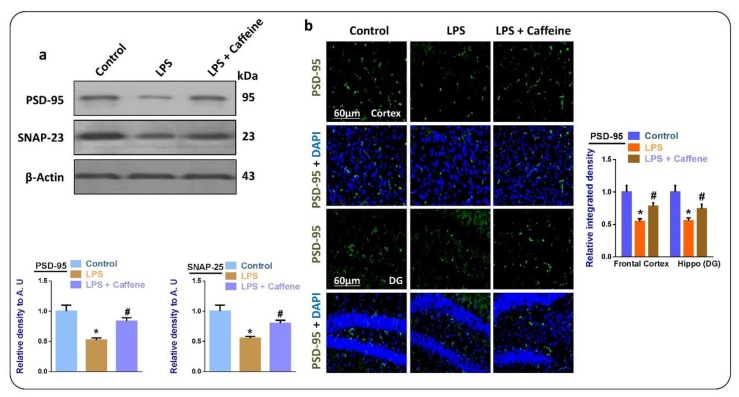
Caffeine inhibits synaptic dysfunction in LPS-injected mouse brains: (**a**) Western blot results of PSD-95 and SANP-23 in the brains of the experimental mice (*n* = 10 mice per group; number of experiments = 3; statistical analysis used was one-way ANOVA with Tukey’s posthoc test for comparisons among the different groups); (**b**) immunofluorescence images of PSD-95 in the experimental groups, magnification 30×, scale bar 60 µm. * significantly different from the vehicle-treated control group, # significantly different from the LPS-treated group. Significance: * *p* < 0.05; # *p* < 0.05. DAPI: 4′, 6-diamidino-2-phenylindole. DG: Dentate gyrus.

We evaluated the effects of caffeine on the synaptic-integrity-related proteins in the LPS-injected mouse brains. For this, we checked the levels of PSD-95 (Postsynaptic density protein 95) and SNAP-23 (Synaptosomal-associated protein 23) in the experimental groups. According to our results, there was reduced expression of PSD-95 and SNAP-23 in the LPS-treated mouse brains. Interestingly, these markers were markedly upregulated in the LPS + caffeine co-treated group (Figure 5a). The immunofluorescence findings further supported the notion that caffeine may inhibit the reduction of the expression of synaptic markers, as here we saw a significant restoration of PSD-95 in the LPS + caffeine co-treated mice (Figure 5b).

## 4. Discussion

Herein, we evaluated the effects of caffeine against LPS-induced oxidative stress, neuroinflammation, apoptotic cell death, and synaptic impairment in LPS-injected mouse brains. According to our findings, there was a significant regulatory effect of caffeine against LPS-induced oxidative stress, neuroinflammation, apoptotic cell death, and synaptic impairment. Currently, the use of phytomedicines is greatly augmented; however, there is still significant research needed before their extensive use and acceptance as neurotherapeutic agents. Extensive research studies have suggested that caffeine, a component of black and green tea, soft drinks, and energy drinks, is the most-consumed psychostimulant [13]. Caffeine exhibits several beneficial properties, including antioxidant [21], anti-inflammatory [22], anti-apoptotic, and neuroprotective effects [23]. Previously, many studies have been conducted on the effects of caffeine; for these, different models have been employed, such as cadmium, amyloid-beta [24], and D-galactose [25]. Moreover, a study was conducted on the protective potential of caffeine against LPS-induced neuroinflammation, where the authors simply focused on the antagonistic effects of caffeine against A1 and A2A adenosine receptors and activated microglia [26]. Their study lacked exploration of the antioxidant and synaptic effects in the LPS-injected mouse model.

To explore the possible antioxidant, anti-inflammatory, and synaptic-related functions of caffeine against the LPS-injected mouse model, we used a six-week treatment schedule. LPS was injected for one week. According to our findings, there were enhanced expression levels of ROS and LPO in the LPS-injected group compared to in the saline-injected control mice. Similarly, the levels of Nrf2 and HO-1 were significantly downregulated in the LPS-injected group. Interestingly, in the LPS + caffeine co-treated groups, these changes were markedly reversed, showing the antioxidant effects of caffeine. These antioxidant outcomes of caffeine are in accordance with previous reports [27]. The downregulation of the expression of Nrf2 and HO-1 are in accordance with the studies conducted previously [28,29], although other studies have reported elevation in the expression of HO-1 with the administration of LPS [30,31].

Next, we analyzed the anti-inflammatory potential of caffeine; for that, we evaluated the levels of different inflammatory markers in the experimental groups. TLR4, which is present at the cell surface of the microglia, plays a pivotal role in the induction of innate immune response, activation of microglial cells, and, ultimately, neuroinflammation [32]. As expected, there was enhanced expression of TLR4, Iba-1, and GFAP in the LPS-treated group. Similarly, p-NF-kB and p-JNK were also evaluated as they are subsequently upregulated in response to activated astrocytes and microglia [33]. Interestingly, the upregulated TLR4 activated microglia and astrocytes, and the upregulated NF-kB and p-JNK were significantly reversed in the LPS + caffeine co-treated group. The activated microglial cells may induce the release of inflammatory mediators, so we checked the relative expression of TNF-α, NOS-2, and Cox-2. In accordance with the expected results, there were enhanced expression levels of TNF-α, NOS-2, and Cox-2 in the LPS-injected group. The upregulation of these markers in the LPS-treated mice is in accordance with previous reports [3]. Interestingly, these markers were markedly reduced in the LPS + caffeine co-treated group.

Mitochondrial apoptosis is a main player in neuronal cell loss [3], so here we examined Bax, Caspase-3, and Bcl-2. According to our Western blot and immunofluorescence studies, there was enhanced expression of Bax and caspase-3 in the LPS-injected group. Interestingly, these markers were significantly reduced in the LPS + caffeine co-treated group, while the level of Bcl-2 was significantly upregulated with the administration of caffeine. The modulatory effects of caffeine against mitochondrial apoptosis have already been reported. Inhibition of the innate immune response and the suppression of apoptotic cell death may be partly due to the inhibition of oxidative stress [34] or directly due to the inhibition of neuroinflammation, as reported previously [35]. Finally, we examined the subsequent synaptic markers, which are associated with the administration of LPS; here, we also found significant restoration of synaptic markers in the LPS + caffeine co-treated group, the same as previously reported in the case of caffeine.

## 5. Conclusions

The overall findings supported the hypothesis that caffeine may potentially regulate oxidative stress, neuroinflammation, and synaptic dysfunctions in LPS-injected mouse brains. The protective effects of caffeine against LPS-induced oxidative damage [36] may be possibly due to it regulating the levels of superoxide dismutase, glutathione peroxidase, and catalase [36,37,38]. Our findings, together with the previous reports [27], suggest that caffeine may reduce elevated oxidative stress, neuroinflammation, and synaptic dysfunction in the LPS-injected mouse brains.
